# Succession, Identity, and Consumption Scale of Prescriptive Ageism: Italian Validation and Invariance by Gender and Age

**DOI:** 10.3390/bs14111027

**Published:** 2024-11-01

**Authors:** Anna Rosa Donizzetti, Cristina Curcio, Michael S. North

**Affiliations:** 1 Department of Humanities, University of Naples Federico II, 80133 Naples, Italy; cristina.curcio@unina.it; 2 Management & Organizations Department, New York University Stern School of Business, New York, NY 10012, USA; msn252@stern.nyu.edu

**Keywords:** ageism, intergenerational tension, scale validation, succession, identity, consumption, SIC of prescriptive ageism, intergenerational-tension ageism scale, invariance

## Abstract

The aim of the work was to achieve the Italian validation of the Succession, Identity, and Consumption Scale of Prescriptive Ageism (SIC) developed by North and Fiske. SIC is a measure of prescriptive ageism, which incorporates intergenerational tensions over practical and symbolic resources. To evaluate the psychometric properties of the Italian version of the scale, two studies were conducted. Study 1 included 931 Italian participants (mean age: 30.94; range: 18–67 years; 50.5% female) and was conducted to test the scale’s structure and construct validity. Study 2 comprised 1015 Italian participants (mean age: 30.73 years; age range: 18–67; 53.5% female) and investigated the scale’s structure, construct validity, and invariance by gender and age. Confirmatory factorial analyses confirmed the three-factor solution to be invariant across sex and age groups. The scale also demonstrated high internal reliability. SIC scores correlated positively with traditional measures for detecting prejudice and stereotypes towards older people. The results of the present work show that the SIC scale of Prescriptive Ageism is a valid tool for measuring prescriptive beliefs about older adults that are the basis of intergenerational tensions.

## 1. Introduction

During the 21st century, we will witness the fastest growth of the worldwide older population ever recorded in human history [1]. This demographic change portends significant social, economic, and political transformations. New challenges will emerge, including nurturing relationships between the various generations, which are affected by pervasive stereotypes about older adults [2]. Thus far, a considerable amount of attention has been directed toward the decline in the health of older people, fueling the perception that the older population represents a burden on society, exacerbating intergenerational tensions and perceptions of scarce available resources [3,4,5].

The term ageism was first introduced by Robert Butler, who described it as “prejudice by one age group toward other age groups” [6] (p. 243). Butler [7] later expanded this definition by incorporating other relevant aspects of the ageism problem, such as the “prejudicial attitudes toward the aged, toward old age, and toward the aging process, including attitudes held by the elderly themselves” (p. 8). Ageism can also manifest in a positive form, whereby individuals are perceived as possessing qualities such as kindness, wisdom, dependability, and affluence [8]. However, negative ageism is more prevalent and has the potential to lead to adverse outcomes. This is because it creates self-fulfilling prophecies [7,9], which can significantly impact social interactions and the quality of life experienced by older individuals [10]. Indeed, individuals internalize negative messages about aging, and this affects their self-perception with a negative impact on their functioning and health, sometimes even decades later [11,12]. Conversely, positive self-perception of aging predicts better functional health [13]. Therefore, combatting prejudices against older people has positive effects not only on older adults today but also on future generations. The prejudice against older adults does not only arise from a negative view of aging, but it can also arise from intergenerational tension due to the perceived scarcity of available resources. This intergenerational tension is exemplified by the perceived divide between Baby Boomers (born between 1946 and 1964) and Millennials (born between 1981 and 1996). Older generations frequently characterize Millennials as lazy, entitled, and disrespectful. On the other hand, Baby Boomers are often criticized by younger generations as being greedy, complacent, and wasteful, accused of exploiting economic, environmental, and political resources to the detriment of other generations. An ageism perspective focused on intergenerational tension highlights this dynamic, emphasizing certain expectations that younger people hold toward older adults. These expectations posit that, having already had their “turn”, older adults are expected to make way for younger generations in various spheres. Young people may have different beliefs about the roles and activities that older people are expected to play within a multi-generational society [14,15]. For example, they may believe that older adults should retire from work as soon as possible to facilitate the succession of enviable assets. They may also advocate for a reduction in the use of healthcare resources to limit the passive consumption of shared goods. Furthermore, they may recommend that older adults refrain from engaging with new technologies to maintain an age-appropriate symbolic identity. This is exemplified by the “Succession, Consumption, and Identity prescriptions” (see below).

## 2. North and Fiske’s Succession, Identity, and Consumption Scale (SIC) of Prescriptive Ageism

While numerous scales have been developed to assess ageism, North and Fiske’s [4] Succession, Identity, and Consumption (SIC) scale is one of the few that specifically targets intergenerational tensions over specific resources. Furthermore, this scale differs from other scales, in that it does not focus on descriptive content concerning the characteristics that older people are presumed to possess. Instead, it employs a prescriptive approach, focusing on expectations concerning the actions that older adults should presumably perform. The prescriptive approach, based on the concept of “ought”, views prejudices and stereotypes as serving to dictate the behavior of other groups in order to benefit the outcomes of the ingroup. Since this aspect of prejudice is more action-oriented, it generates greater differences between groups compared to descriptive prejudices, which merely capture the characteristics of the outgroup. This tendency can lead to even more negative consequences when applied to the existing opposition between younger people and older people in managing society’s limited resources. Young people, feeling deprived by older adults of the resources they believe are rightfully theirs, strongly endorse prescriptive stereotypes [4]. Ageism measures have largely overlooked these prescription-based possibilities. The SIC scale has been developed with the specific aim of addressing this limitation of existing scales.

This measurement instrument thus focuses on the idea that age groups are interdependent, thereby fostering intergenerational tensions with respect to practical and symbolic resources. Despite the existence of psychosocial theories that are useful for understanding and managing these dynamics, such as the Realistic Group Conflict Theory [16], little is performed for intergenerational tensions arising from resource scarcity [17,18]. The SIC scale refers to three domains of intergenerational tension over the distribution of resources: facilitating the active, intergenerational succession of enviable resources (e.g., retiring from work, surrendering economic resources), limiting the passive consumption of shared resources (e.g., minimizing healthcare expenditures, speeding up motorway traffic), and avoiding the appropriation of those symbolic resources typically associated with youth identity (e.g., popular music, fashion). The SIC consists of 20 items, measured on a 6-point Likert scale, ranging from 1 = strongly disagree to 6 = strongly agree. The items are divided into three dimensions: Succession (8 items), Consumption (7 items), and Identity (5 items). North and Fiske [4] used four samples (with a total of 2010 participants) to validate the scale. The samples were from the United States and aged between 16 and 81 years. Starting with a list of 41 items, the authors arrived at a 20-item, 3-factor solution by means of exploratory factor analysis, a result that was subsequently validated by confirmatory factor analysis. This solution was also confirmed with the other three samples. The Cronbach’s alpha coefficients in the four samples ranged from 0.90 to 0.91 for the total scale, while for the three subscales, the values varied: from 0.84 to 0.85 for Succession, from 0.83 to 0.87 for Identity, and from 0.75 to 0.86 for Consumption. 

Hancock and Talley [19] subsequently sought to conduct a more detailed examination of the scale’s reliability, factorial structure, and invariance by gender and ethnic group of this scale. The participants (N = 1790) were 69% female and 69% identified as White. Furthermore, 60% of the participants were between the ages of 18 and 24 years, 33% were between 25 and 34 years, and 7% were 35 years of age or older. The three-factor factorial structure was confirmed (χ^2^ = 2471.78, df = 164; RMSEA = 0.09; CFI/TLI = 0.93/0.91). However, the modification indices suggested a better fit of the model to the data by moving the item “Older people shouldn’t be so miserly with their money if younger relatives need it” from the subscale of Consumption to the subscale of Succession. In addition, the item “Older people probably shouldn’t use Facebook” was removed from the alternative factor model due to low and insignificant coefficients. The fit indices of the alternative model were better and exceeded the limits of acceptability (RMSEA = 0.08; CFI/TLI = 0.94/0.93). Invariance analyses were conducted on the alternative factorial model. Chi-square difference tests between the configural and scalar models were significant, indicating non-invariance of the measure with respect to both sex and ethnic subgroups. Cronbach’s alpha coefficients demonstrated satisfactory reliability, with values of 0.84 for Succession, 0.78 for Identity, and 0.78 for Consumption.

More recently, Boudjemadi et al. [20] examined the generalizability of the SIC scale in four French-speaking countries, namely, Canada, France, Belgium, and Switzerland. The research aimed to test the factorial validity, construct validity, and reliability of the SIC scale for each country as well as to assess the multi-group invariance of the SIC scale between the countries involved. Three items were excluded a priori: the item “AARP (American Association of Retired Persons) wastes charity money” as there is no equivalent association in France, Belgium, Canada, and Switzerland; the item “Most older workers don’t know when it’s time to make way for the younger generation” and the item “Older people typically shouldn’t go to places where younger people hang out” because they were identified as highly redundant with the other items in the original study. Moreover, in accordance with the findings of Hancock and Talley’s study [19], the authors replaced “social network” with “Facebook” in the item “Older people probably shouldn’t use Facebook”. Following confirmatory factor analyses, two items were found to be problematic, both from the Consumption subscale. The first was “Older people shouldn’t be so miserly with their money if younger relatives need it”. In each sample, this item scored on its reference factor unacceptable. The second was “Older people don’t really need to get the best seats on buses and trains”, which exhibited low factor loadings in Switzerland and Belgium, while in France and Canada, it showed strong correlations with several subscale items. The 15-item scale shows good fit indices (RMSEA = 0.044 in France, 0.039 in Canada, 0.041 in Switzerland, and 0.045 in Belgium; CFI/TLI = 0.94/0.93 in France, 0.94/0.92 in Canada, 0.94/0.92 in Switzerland, and 0.91/0.89 in Belgium). In addition, the data also fit a second-order factor (RMSEA = 0.071 in France, 0.45 in Canada, 0.69 in Switzerland, and 0.68 in Belgium; CFI/TLI = 0.84/0.81 in France, 0.91/0.90 in Canada, 0.82/0.79 in Switzerland, and 0.80/0.76 in Belgium). For both models, the results provided evidence of configural invariance, weak factorial invariance, and partial strong factorial invariance. The analyses demonstrated excellent reliability in the samples from all four countries, with Rho scores ranging from 0.84 in the Swiss sample to 0.92 in the Canadian sample for the total scale, while for the three subscales, the values ranged from 0.65 to 0.74 for Succession, from 0.64 to 0.76 for Identity, and from 0.61 to 0.73 for Consumption.

## 3. Review of Studies That Have Used the SIC Scale

The studies that have used the SIC scale to detect ageism are diverse and in disparate subject areas, such as health, workplaces, socio-political decisions, and intervention.

More recent studies have elucidated ageism’s health-level impacts. In the domain of physical and mental health, ageism has been linked to a number of adverse outcomes, including an elevated risk of developing post-traumatic stress syndrome [21] and cognitive disorders [22]. Furthermore, negative effects have also emerged concerning the will to live, especially among older adults, particularly when their health deteriorates [23]. Studies conducted during the COVID-19 pandemic showed a significant correlation between ageism and COVID-19-related health concerns [24], as well as an increased fear of contracting the virus [25].

The workplace is another crucial domain in which intergenerational tensions are prevalent. The perception of a scarcity of resources within workplaces reduces the likelihood of younger workers engaging in networking with older workers. Furthermore, when workers are asked to distribute scarce training resources among three similarly qualified but differently aged employees (young, middle-aged, and older), older workers receive the lowest investment [26].

In the socio-political sphere, a positive relationship emerges between advocacy of egalitarianism and ageism. Specifically, the more participants support the concept of equality, the more they believe that older individuals should actively step aside. Political liberalism was also correlated with greater ageism, as was being less resistant to societal change to create equality [27]. Another study [28] shows that a more conservative political orientation is also associated with a more negative attitude toward older people. 

The measurement of ageism through an intergenerational conflict lens has the potential to serve as a useful barometer for the reduction of prejudice. In a study by Lytle and Levy [29], participants randomly assigned to the experimental group had to watch three videos (less than 10 min in total) in which stereotypes about aging and older adults were challenged, positive intergenerational contact was portrayed and older adults were highlighted as being able to make a positive contribution to society; by contrast, participants in the control group watched three neutral videos. Watching videos that positively portray older adults led to a reduction in intergenerational conflict. Another study [30] sought to ascertain the impact of a gerontology course on the attitudes of nursing students toward older people, measuring changes in attitudes toward ageism and other related dimensions. Having benefited from ad hoc training on aging and older people resulted in a significant decrease in ageism. Likewise, Au et al. [31] used an intergenerational mentoring program for university students in which non-fragile older people were paired with students to provide support to frail older adults. This facilitated a reduction in intergenerational tension.

## 4. Aim and Hypotheses

The aim of this study is to validate the Succession, Identity, and Consumption Scale of Prescriptive Ageism (SIC) in an Italian sample. This scale is capable of detecting ageism as an expression of intergenerational tension. In addition to supporting the SIC scale’s generalizability to a different context, this endeavor is motivated by pragmatic concerns: Italy is one of the industrialized nations with the highest rate of population aging. This necessitates a valid instrument that can be used for both research purposes and interventions aimed at reducing prejudice based on intergenerational contact. This validation is based on two studies examining the structure, reliability, convergent validity, and invariance across gender and age groups. We hypothesized that the SIC scale is three-dimensional and would have high internal reliability (Studies 1 and 2).

We also hypothesized that the SIC scale would demonstrate good convergent validity by positively correlating with corresponding measures of ageism (specifically, the Fraboni Scale of Ageism and the Ageing Semantic Differential; Studies 1 and 2). In particular, the SIC and the FSA are both scales measuring ageism. However, the latter is primarily concerned with the characteristics and attributes ascribed to older individuals, thereby focusing on descriptive stereotypes. In contrast, the SIC scale is concerned with the role of prescriptive beliefs based on control and the notion of “should” [4].

With regard to group invariance (Study 2), we hypothesized that the functioning of the SIC items would not differ across the gender and age groups, in line with prior findings.

## 5. Materials and Methods

### 5.1. Adaptation of the Original Version SIC into Italian

The original version of the SIC scale was translated into Italian using a cross-cultural adaptation methodology through a multi-step procedure. 

Translation and back-translation. The SIC was initially translated into Italian and subsequently back-translated into English to ensure translation equivalence. Two bilingual PhDs were involved in the translation process. An Italian social psychology researcher translated the original instrument into Italian. Another native English-speaking researcher, who is also an expert in social psychology, translated the SIC from Italian into English, without any comparison with the initial translator. 

Committee review. Subsequently, a group of experts composed of the two researchers responsible for the translations, along with two additional social psychology researchers compared the two versions of the scale and assessed the accuracy of the translation, making the necessary adjustments to ensure comprehensibility, adaptation to the specificities of the context, and psychological equivalence. In the opinion of the review committee, the original version and the retranslated Italian version did not differ appreciably.

Committee for content validity. Before starting the study, the instrument was submitted to a panel of experts to establish its content validity (face validity). This expert panel consisted of three academic researchers, who successfully validated the measure.

Pre-test. Finally, a convenience sample of 15 people of different ages was used to test the questionnaire. Participants were contacted and asked to provide feedback on the item content, which was used to improve the wording of the questionnaire items [32] (see Table 1).

Finally, we proceeded to administer the instrument to test the psychometric properties of the Italian version of the SIC.

### 5.2. Procedure and Statistical Analyses (Studies 1 and 2)

Participants in the two studies were recruited through snowball sampling. Students of two university courses—one at the end of 2020 (September–December) and the other at the end of 2021 (December)—were asked to complete a questionnaire and to forward the questionnaire, implemented on the Google Forms platform, to other subjects by word of mouth. Participation in the study was voluntary and anonymous. Participants were encouraged to answer as truthfully as possible and were informed that they could withdraw from the study at any time. The study protocol was approved by the local ethics committee (Ethical Committee of Psychological Research of the Department of Humanities of the University of Naples Federico II, prot. 10/2019), and the study was conducted in accordance with the ethical standards set out by the American Psychological Association. The study complied with the ethical principles of the 1995 Declaration of Helsinki. Participants gave consent to participate on the first page of the survey, which took approximately 30 min to complete.

Data analysis was performed using the statistical software SPSS 28.0 [33] and M-PLUS 8.0 [34]. After collecting the questionnaires, we performed matrix cleaning, response frequency analysis, and exploratory factor analysis. To test the structural validity, we also performed confirmatory factor analysis (CFA) using the Maximum Robust Likelihood (MLR), which provides accurate estimates in the presence of non-normal distributions [35]. The fit indices used in this study are as follows: chi-squared distribution and degrees of freedom (χ^2^/df), Comparative Fit Index (CFI), Tucker–Lewis Index (TLI), Root Mean Square Error of Approximation (RMSEA), and Standardized Root Mean Square Residual (SRMR). According to the recommendations of McDonald and Ho [36] and Hu and Bentler [37], the χ^2^/df should be in a range between 2 and 5; the values of the CFI and of the TLI must be >0.90; those of RMSEA are considered to be good if they are <0.05, reasonable if they are <0.08, and average if they are <0.10 [38]; those of SRMR must be <0.09 [39]. Cronbach’s alpha was calculated in order to check the internal consistency of the scale (values greater than 0.70 are significant [40]). In addition, internal consistency was also assessed by calculating the correct correlation between the item score and the total scale. The convergent and discriminant validity was verified by means of Pearson’s correlation analysis (*p*-value < 0.05).

Invariance decisions were made by considering changes in CFI, RMSEA, and SRMR. Invariance was confirmed if the change in CFI was less or equal to 0.010, the one in RMSEA was less than 0.015, and a change in SRMR less or equal to 0.030 was considered as the threshold for testing metric invariance, and less or equal to 0.010 for assessing scalar invariance [41,42].

### 5.3. Measures

Succession, Identity, and Consumption Scale of Prescriptive Ageism (SIC) [4] consists of 20 items rated on a six-point Likert scale, ranging from 1 (strongly disagree) to 6 (strongly agree). Thus, higher scores indicate higher levels of intergenerational tension.

Fraboni Scale of Ageism. The Fraboni Scale of Ageism (FSA) [43,44] assesses, through 19 statements (4-point Likert scale, ranging from 1 = strongly disagree to 4 = strongly agree), the cognitive and affective components of ageism. Higher scores indicate greater levels of ageism. In this study, the internal consistency reliability of the scale (Cronbach’s α) was 0.76 (Study 1) and 0.77 (Study 2).

Aging Semantic Differential. Aging Semantic Differential (ASD) [45,46,47] measures the impact of stereotypes on respondents’ attitudes toward older adults. Twenty pairs of opposing adjectives are used on a seven-point semantic differential scale. Higher scores indicate greater levels of stereotypical views. The Cronbach’s α of the scale was 0.92 (Study 1 and Study 2).

Additionally, a socio-demographic data sheet was included.

## 6. Study 1

### 6.1. Participants

A total of 931 Italian participants took part in the first study (50.5% female, 49.5% male), aged between 18 and 67 years (M = 30.94, SD = 15.29). About 68.0% were university students, 13.2% employees, 10.2% freelancers, 7.4% unemployed, and 1.2% retired. In addition to the 68.0% still in education, 14.9% had a high school diploma, 7.4% had a university degree, 6.2% with a secondary school diploma, 2.7% had a postgraduate degree, and a marginal 0.8% had a primary school diploma. 

### 6.2. Results

#### 6.2.1. Descriptive Analyses

In order to examine the quality of the items and the probability of dysfunctional or biased items, we estimated the variances, means, and standard deviations of the 20 items of the SIC. The results shown in Table 2 indicate that all items have a normal distribution in terms of sample responses, with the exception of items 4 (Asymmetry = 1.235 and Kurtosis = 1.526), 16 (Asymmetry = 1.522 and Kurtosis = 2.580), and 18 (Asymmetry = 1.205 and Kurtosis = 2.018). However, severe skewness (>3 in absolute value) and kurtosis (>10 in absolute value) are not present. Therefore, the distribution is likely not severely non-normal [48]. The item–test correlations are between 0.27 and 0.60, and although item 13 has a low item–total correlation (0.27), its removal would not lead to an increase in Cronbach’s alpha.

#### 6.2.2. Confirmatory Factor Analysis of the Factorial Structure of SIC Scale

A confirmatory factor analysis was performed by testing the three-dimensional structure that emerged in North and Fiske’s study [4]. The CFA results did not yield satisfactory fit indices [χ^2^/df = 891.282 (164), *p* ≤ 0.001; χ^2^/df = 5.43; CFI = 0.882; TLI = 0.863; RMSEA = 0.069 (0.065–0.063); and SRMR = 0.059].

#### 6.2.3. Exploratory Factor Analysis and Confirmatory Factor Analysis of the Italian Version of the SIC Scale

Due to the numerous modification indexes, we decided to proceed with an exploratory factor analysis, which initially revealed a four-factor structure with eigenvalues greater than 1. The Bartlett sphericity test was significant: χ^2^ (190) = 6286.353 (*p* ≤ 0.000), as was the Kaiser–Meyer–Olkin sample adequacy test, which was 0.898. A three-factor solution was extracted based on the scree plot and in accordance with the reference literature. The first item to be eliminated was item 13 (“Older people shouldn’t be so miserly with their money if younger relatives need it”) which had high saturations on all three factors and also had the lowest item–total correlation score in the previous phases of the analyses. The second item to be eliminated was item 12 (“Older people probably shouldn’t use Facebook”), which did not have satisfactory saturations on any of the three dimensions. Finally, the third item eliminated was item number 2 (“If it weren’t for older people opposed to changing the way things are, we could probably progress much more rapidly as a society”) as it had high saturations on two of the three dimensions. The factorial solution with 17 items distributed in three factors (Bartlett’s sphericity test: χ^2^ (136) = 5306.634 (*p* ≤ 0.000); Kaiser–Meyer–Olkin: 0.887) explained 42.51% of the total variance. The three factors overlap in content with the dimensions of Succession, Consumption, and Identity as described by the reference authors [4] (Table 3).

The 17-item trifactorial structure was subjected to confirmatory factor analysis and showed excellent fit indices [χ^2^/df = 361.843 (113), *p* ≤ 0.000; χ^2^/df = 3.20; CFI = 0.939; TLI = 0.927; RMSEA = 0.049 (0.043–0.054); and SRMR = 0.043]. The standardized coefficients for the Succession dimension range from 0.52 to 0.73, for the Consumption dimension from 0.50 to 0.70, and for the Identity dimension from 0.61 to 0.76. Additionally, error correlations between SIC 6 and SIC 3 (0.41), SIC 11 and SIC 8 (0.35), and SIC 1 and SIC 4 (0.14) were confirmed, consistent with the original validation.

#### 6.2.4. Concurrent Validity

The results show that SIC and its subscales correlate positively and significantly with all variables considered in this study (Table 4).

## 7. Study 2

### 7.1. Participants

About 1015 Italians participated in the second study (53.5% female, 46.5% male), aged between 18 and 67 years (M = 30.73, SD = 14.90): 67.2% were university students, 14.8% employed, 8.2% were freelancers, 8.3% were unemployed, and 1.5% were retired. In addition to the 67.2% still in education, 15.4% were high school graduates, 7.0% were university graduates, 6.8% had a secondary school degree, 2.2% had a postgraduate degree, and 1.4% had a primary school degree. 

### 7.2. Results

#### 7.2.1. Descriptive Analyses

The quality of the items was checked by estimating the variances, means, and standard deviations of the 17 items of the SIC. The results shown in Table 5 indicate that all items have a normal distribution based on sample responses, except for items 4 (Asymmetry = 1.239 and Kurtosis = 1.502), 16 (Asymmetry = 1.414 and Kurtosis = 2.277), and 18 (Asymmetry = 1.157 and Kurtosis = 1.735). Severe skewness (>3 in absolute value) and kurtosis (>10 in absolute value) are not present for this sample, indicating that the distribution is likely not severely non-normal [48]. The item–test correlations are between 0.33 and 0.61. 

#### 7.2.2. Confirmatory Factor Analysis of the Factorial Structure of the Italian Version of the SIC Scale

The 17-item trifactorial model was subjected to confirmatory factor analysis. The CFA results show good fit indices [χ^2^/df = 439.743 (113), *p* ≤ 0.000; χ^2^/df = 3.89; CFI = 0.932; TLI = 0.918; RMSEA = 0.053 (0.048–0.059); and SRMR = 0.049]. The standardized coefficients of the dimension Succession range from 0.51 to 0.77; those of the dimension Consumption from 0.45 to 0.73; and those of the dimension Identity from 0.51 to 0.81. The error correlations between items SIC 6 and SIC 3 (0.32), SIC 11 and SIC 8 (0.32), and SIC 1 and SIC 4 (0.30) were also confirmed, consistent with the original validation. 

#### 7.2.3. Concurrent Validity

Also in the second study, SIC and its subscales correlated positively and significantly with all variables considered in this study (Table 6).

#### 7.2.4. Analysis of Invariance by Gender and Age of the Italian Version of SIC Scale

In order to verify the invariance of gender and age, the three-factor correlated model was tested in the individual groupings and subsequently in a simultaneous manner. In particular, the multiple-group analysis comprises three distinct forms of invariance testing: configural, metric, and scalar. In order to test for configural invariance, the model is estimated simultaneously in the two groups without any constraints being placed on it. In order to test for metric invariance, the model is estimated simultaneously in the two groups, with the saturations constrained. Finally, for scalar invariance, the model is estimated simultaneously in the two groups by constraining the saturations and variance-covariances between factors. 

The analyses conducted with respect to gender demonstrated that the models tested separately in the male (N = 472) and female (N = 543) groups exhibited satisfactory fit indices. Moreover, the results of the simultaneous analyses with varying degrees of constraint demonstrated that all assumptions of invariance were validated (Table 7).

Subsequently, the hypothesis of age invariance was tested by dividing the reference population into two distinct groups: young people (range: 18–35; N = 676) and adults (range: 36–67; N = 316). Once more, the model was tested in the two subsamples separately. The model demonstrated an excellent fit to the data in each group (Table 8).

The multi-group invariance analysis by age demonstrated that the metric invariance was confirmed.

## 8. Discussion and Conclusions

The rapid shifts in age demographics have underscored the imperative for a reassessment of existing policies, with a view to transforming perceptions of the older population from a liability to an asset. This need, however, clashes with persistent stereotypes and generational tensions. For example, older workers generally hold enviable job positions and are thus at greater risk of being seen as obstacles to younger people’s professional goals [18]. To effectively intervene in these dynamics, it is necessary to first be able to measure them. The SIC scale has yielded significant findings spanning various fields, from the socio-political [27,28] and occupational [26] to physical and mental health [21,22,23] and prejudice reduction [29,30,31]. The distinctive feature of this instrument is its ability to detect ageism not by referring to descriptive aspects and thus to what older people should be like, but through a prescriptive approach, referring to expectations regarding what older adults should do [4].

This study aimed to achieve the Italian validation of the SIC scale for detecting intergenerational tensions. The final solution consists of 17 items; in fact, exploratory factor analysis eliminated three items, one for each of the dimensions that emerged. Items 13 and 12 had also shown problems in previous validations [19,20]. Conversely, item 2 was identified as a potential issue solely within the context of the Italian sample. The content of this item likely evoked both aspects related to the role older adults play in preventing resource succession—such as the transition from the old to the new—and aspects related to consumption, reflecting the desire to maintain conditions of advantage that change could potentially undermine. This result may be due to the specific demographic configuration of Italy, which sets it apart from all other European countries. Over the past 50 years, Italy has experienced one of the fastest aging processes among developed countries, recording the highest average population age in Europe [49]. These data raise important questions about the country’s ability to manage the limited economic resources available [50] to cope with a demographic reality never experienced by any large nation and to provide quality services to such a broad and steadily increasing segment of the population. In this context, the country faces the challenge of balancing resource distribution to ensure quality care for this age group while also addressing the needs of other age groups. It is within this framework that the discussion on the specificities of the existing intergenerational tensions in Italy and the potential use of the SIC scale to understand this ongoing demographic change must be positioned.

In relation to the factorial structure, it should also be highlighted that there were no correlations between the errors different from those found in the original study. Moreover, the findings of the two provided substantial evidence for the reliability of this instrument, indicating the stability of SIC’s three-dimensional structure across different sex and age groups, and confirming its convergent validity. 

The present work has the merit not only of having confirmed the stability of the factorial structure in the Italian context but also of having tested invariance by age, which had not been previously explored, and which instead is particularly useful considering the subject matter. Having arrived at the Italian validation of the SIC scale will also make it possible to initiate cross-cultural studies that will allow us to capture the common and distinctive aspects of this phenomenon in different contexts. Furthermore, the scale, by providing valuable information about individuals’ perceptions of older people, is particularly useful in settings where actions to reduce ageism need to be implemented.

Although the results of these two studies are extremely encouraging, the present research has some limitations that future studies could address. First, by using a self-report questionnaire, self-reported data could lead to common method variance problems. Therefore, it would be important to test associations between the SIC scale and actual behavioral measures, to also consider non-self-reported ratings as well. Our study certainly has the limitation of considering only one measure of explicit self-reported ageism. It would be interesting in the future to consider other measures of ageism, such as those that focus on the social and relational nature of prejudice, like discursive psychology [51], rhetorical social psychology [52], and conversation analysis [53]. These studies regard evaluative discourse as a set of linguistic acts [51], analyze the construction of the object of evaluation, and study the communicative practices through which evaluations are made [54]. In discursive social psychology, attitudes involve making evaluations or taking stands on controversial issues [52], and ageism and intergenerational tensions certainly fall into this category. In everyday argumentation, people do not merely take stands on controversial issues; they also justify their positions. A simple focus on positions overlooks these justifications, losing part of the meaning. Other tools that could be useful are those that assess implicit prejudice, among which the most well-known is the Implicit Association Test (IAT), which measures response times to faces of young and older individuals in association with words of positive and negative valence [55].

Furthermore, the implementation of a longitudinal study would allow for more reliable model testing over time and for test–retest calculations. Validity tests were conducted on convenience samples that are not fully representative of the general population, and future research should test whether this result is reproducible in a representative, more age-balanced sample. In fact, although an age invariance analysis was conducted, the sample is predominantly composed of students, and this could affect the generalizability of the results. A further limitation is the lack of consideration for objective indicators of intergenerational tension in the validation process, such as the actual exclusion of older adults from certain environments reserved exclusively for young people, like nightclubs, or the allocation of fewer job promotions to older individuals compared to younger ones, or even the less time that doctors spend with older patients compared to younger ones. In addition, self-report instruments are potentially subject to social desirability bias problems. Although it is necessary to consider this limitation, it is reasonable to assume that our data are not highly influenced by this bias because anonymity was guaranteed during the data collection process [56]. 

In conclusion, the use of the SIC scale should be recommended not only because of its excellent and stable psychometric properties but also because of this instrument’s ability to capture the distinctiveness of ageism compared to other isms. Age, in fact, is one of the primary categories through which we categorize people, but it is also the only universal category in that everyone who lives long enough will take part in it [18]. Therefore, ageism by anchoring itself to an evolving category is dynamic rather than static, unlike other categories such as race or gender. The value of this instrument lies in its ability to capture this dynamism as opposed to classical instruments by focusing on what older adults are supposed to do and not on perceptions of how older people supposedly are [4].

Considering the robust psychometric properties of the scale, we recommend a broad use of SIC as a whole, but also in relation to its specific dimensions. For example, the succession dimension may find application in capturing the dynamics of ageism in the workplace and the contemporary retirement age debate. The consumption dimension, on the other hand, plays a key role in current debates on health care. The subscale of identity can make a contribution to the theorization of ageism within the framework of Social Identity Theory [57,58], whereby ageism might derive from the tendency of young people to favor the ingroup, but also in more applied domains, concerning for instance the need for companies to market products that are symbolically suitable for the older population. 

The practical implications of using the SIC scale could involve its interesting application within workplace contexts to measure the levels of intergenerational tension existing among colleagues or between managers and employees. This could help, for instance, in understanding whether there are indeed disparities in access to promotions, career advancements, or even training and professional development opportunities. Another interesting application context could be in healthcare, to better understand the relationships between healthcare providers and older patients, to provide tools for improving service quality for patients of all ages. The use of the SIC scale could also encourage reflection on access to the social and political life of the country, to understand how existing intergenerational tensions may influence each person’s contribution. These reflections would be particularly relevant in the Italian context, which, as we have seen, is characterized by a significant presence of older individuals and a scarcity of public resources that various social actors are forced to share with difficulty. Therefore, understanding how intergenerational tensions exacerbate the already existing challenges in areas such as employability and healthcare would offer a new perspective on the issue. In Italy, the oldest country in Europe [49], population aging presents major challenges regarding the availability of economic and social resources across generations. Recent events have already made these tensions salient. For example, during the COVID-19 pandemic when resources for the health sector were in short supply, older adults reported the highest levels of perceived age discrimination, and these perceptions exacerbated their feelings of loneliness [59]. This sentiment negatively affects confidence in the future positive affect [60], which is a crucial element in maintaining healthy and active aging. Similarly, in the workplace, the experience of age discrimination has been linked to reduced job satisfaction and a reduced likelihood of changing organizations [61]. An instrument that focuses on expectations of what older people should do is particularly useful in contexts characterized by high levels of generational tension. Our hope is that the Italian validation will significantly contribute to this ongoing and increasingly important research conversation.

## Figures and Tables

**Table 1:** English and Italian versions of the items.

English Version	Italian Version
Doctors spend too much time treating sickly older people. *	1. I medici passano troppo tempo a curare persone anziane malate.
Older people are too big a burden on the healthcare system. *	4. Le persone anziane sono un peso troppo grande per il sistema sanitario.
Older people are often too much of a burden on families.	7. Le persone anziane sono spesso un carico eccessivo per le famiglie.
At a certain point, older people’s maximum benefit to society is passing along their resources.	10. A un certo punto, il massimo beneficio che le società possono trarre dalle persone anziane è il trasferimento delle loro risorse.
Older people shouldn’t be so miserly with their money if younger relatives need it.	13. Le persone anziane dovrebbero aiutare con la loro diponibilità economica se i parenti più giovani ne hanno bisogno.
Older people don’t really need to get the best seats on buses and trains.	16. Le persone anziane non hanno davvero la necessità di avere i posti migliori su autobus e treni.
AARP (American Association of Retired Persons) wastes charity money.	18. Sostenere con azioni di beneficenza le associazioni per anziani è uno spreco di denaro.
If it weren’t for older people opposed to changing the way things are, we could probably progress much more rapidly as a society.	2. Se non fosse per gli anziani che si oppongono al cambiamento, probabilmente potremmo progredire molto più rapidamente come società.
The older generation has an unfair amount of political power compared to younger people.	5. La vecchia generazione ha un’ingiusta quantità di potere politico rispetto ai giovani.
Most older people don’t know when to make way for younger people. ^+^	8. La maggior parte delle persone anziane non sa quando lasciare il post ai giovani.
Most older workers don’t know when it’s time to make way for the younger generation. ^+^	11. La maggior parte dei lavoratori più anziani non sa quando è il momento di lasciare il posto alle nuove generazioni.
Older people are often too stubborn to realize they don’t function like they used to.	14. Le persone anziane sono spesso troppo testarde per rendersi conto che non hanno più le funzionalità di una volta.
Younger people are usually more productive than older people at their jobs.	17. I giovani di solito sono più produttivi degli anziani nel loro lavoro.
Job promotions shouldn’t be based on older workers’ experience rather than their productivity.	19. Le promozioni di lavoro non dovrebbero basarsi sull’esperienza dei lavoratori più anziani piuttosto che sulla loro produttività.
It is unfair that older people get to vote on issues that will impact younger people much more.	20. Non è giusto che le persone anziane possano votare su questioni che avranno un impatto maggiore sui giovani.
Older people typically shouldn’t go to places where younger people hang out. ^x^	3. Le persone anziane in genere non dovrebbero andare nei luoghi in cui i giovani si incontrano.
In general, older people shouldn’t hang out at places for younger people. ^x^	6. In generale, le persone anziane non dovrebbero frequentare posti per i giovani.
Generally older people shouldn’t go clubbing.	9. Generalmente le persone anziane non dovrebbero andare in discoteca.
Older people probably shouldn’t use Facebook.	12. Le persone anziane probabilmente non dovrebbero usare Facebook.
Older people shouldn’t even try to act cool.	15. Le persone anziane non dovrebbero nemmeno provare a comportarsi in modo cool.

Note: * ^+ x^ Similar items denoted as co-varying in the structural equation model.

**Table 2:** Mean, standard deviations, variance, asymmetry, kurtosis, corrected correlations, and reliability of the item (Study 1).

	Mean	Standard Deviation	Variance	Asymmetry	Kurtosis	Corrected Correlation	α If Item Deleted
SIC 1	2.14	1.15	1.32	1.018	0.783	0.381	0.88
SIC 2	2.84	1.44	2.07	0.505	−0.610	0.537	0.87
SIC 3	2.26	1.15	1.33	0.946	0.815	0.544	0.87
SIC 4	2.01	1.12	1.25	1.235	1.526	0.549	0.87
SIC 5	3.53	1.46	2.13	0.050	−0.887	0.442	0.88
SIC 6	2.30	1.14	1.30	0.850	0.477	0.579	0.87
SIC 7	2.32	1.14	1.29	0.689	−0.013	0.552	0.87
SIC 8	3.56	1.30	1.70	−0.167	−0.467	0.604	0.87
SIC 9	3.17	1.48	2.19	0.245	−0.882	0.464	0.88
SIC 10	2.50	1.27	1.62	0.751	0.116	0.540	0.87
SIC 11	3.62	1.33	1.76	−0.143	−0.572	0.592	0.87
SIC 12	2.45	1.36	1.85	0.977	0.325	0.481	0.88
SIC 13	4.02	1.14	1.29	−0.379	0.101	0.272	0.88
SIC 14	3.83	1.29	1.66	−0.383	−0.212	0.545	0.87
SIC 15	2.34	1.18	1.39	0.793	0.347	0.469	0.88
SIC 16	1.86	1.05	1.11	1.522	2.580	0.348	0.88
SIC 17	3.71	1.26	1.58	−0.228	−0.405	0.422	0.88
SIC 18	1.92	0.96	0.92	1.205	2.018	0.452	0.88
SIC 19	3.50	1.37	1.87	0.040	−0.771	0.423	0.88
SIC 20	3.06	1.40	1.95	0.335	−0.619	0.577	0.87

**Table 3:** Exploratory factor analysis, and membership factor of each item in the validations of North and Fiske, Hancock and Talley, and Boudjemadi and colleagues.

	**F1**	**F2**	**F3**	**Item–Total Corr.**	**Alpha If the Item Is Deleted**	**North and Fiske**	**Hancock and Talley**	**Boudjemadi and Colleagues**
**% Variance**	29.98	8.25	4.29					
**α**	0.81	0.78	0.78					
SIC 11	0.81	−0.05	−0.03	0.66	0.77	S	S	R
SIC 8	0.81	0.03	−0.08	0.68	0.77	S	S	S
SIC 14	0.57	0.01	0.09	0.55	0.79	S	S	S
SIC 5	0.54	0.02	−0.04	0.48	0.80	S	S	S
SIC 19	0.53	−0.04	0.03	0.49	0.80	S	S	S
SIC 17	0.52	−0.10	0.09	0.46	0.80	S	S	S
SIC 20	0.50	0.12	0.08	0.55	0.79	S	S	S
SIC 4	0.06	0.75	−0.09	0.63	0.71	C	C	C
SIC 18	−0.10	0.65	0.08	0.54	0.74	C	C	R
SIC 16	−0.12	0.59	0.01	0.44	0.76	C	C	R
SIC 1	−0.02	0.56	−0.02	0.47	0.76	C	C	C
SIC 10	0.17	0.51	0.02	0.54	0.74	C	C	C
SIC 7	0.20	0.46	0.06	0.52	0.74	C	C	C
SIC 6	−0.03	0.03	0.85	0.69	0.67	I	I	I
SIC 3	−0.07	0.14	0.72	0.63	0.70	I	I	R
SIC 9	0.18	−0.18	0.64	0.53	0.76	I	I	I
SIC 15	0.01	0.16	0.47	0.51	0.76	I	I	I
SIC 13	-	-	-	-	-	C	S	R
SIC 12	-	-	-	-	-	I	R	I
SIC 2	-	-	-	-	-	S	S	S

Note: S: Succession; C: Consumption; I: Identity; R: Removed.

**Table 4:** Correlational analysis (Study 1).

	1	2	3	4	5	6
1. SIC	1					
2. SIC_Succession	0.85 **	1				
3. SIC_Consumption	0.79 **	0.45 **	1			
4. SIC_Identity	0.75 **	0.43 **	0.53 **	1		
5. FSA	0.60 **	0.38 **	0.61 **	0.51 **	1	
6. ASD	0.21 **	0.18 **	0.20 **	0.14 **	0.24 **	1

Note: ** *p* < 0.01.

**Table 5:** Mean, standard deviations, variance, asymmetry, kurtosis, corrected correlations, and reliability of the item (Study 2).

	Mean	Standard Deviation	Variance	Asymmetry	Kurtosis	Corrected Correlation	α If Item Deleted
SIC 1	2.17	1.177	1.385	0.979	0.550	0.35	0.86
SIC 3	2.25	1.102	1.214	0.887	0.700	0.59	0.85
SIC 4	2.01	1.106	1.224	1.239	1.502	0.58	0.85
SIC 5	3.71	1.511	2.285	−0.047	−0.993	0.47	0.86
SIC 6	2.33	1.091	1.191	0.878	0.755	0.60	0.85
SIC 7	2.48	1.176	1.382	0.578	−0.231	0.55	0.86
SIC 8	3.61	1.374	1.889	−0.049	−0.770	0.61	0.85
SIC 9	3.00	1.457	2.123	0.459	−0.690	0.47	0.86
SIC 10	2.48	1.209	1.461	0.638	−0.150	0.55	0.86
SIC 11	3.68	1.361	1.852	−0.088	−0.708	0.59	0.85
SIC 14	3.87	1.298	1.685	−0.253	−0.432	0.47	0.86
SIC 15	2.31	1.114	1.242	0.908	0.743	0.38	0.86
SIC 16	1.86	1.004	1.009	1.414	2.277	0.33	0.86
SIC 17	3.83	1.226	1.502	−0.175	−0.363	0.47	0.86
SIC 18	1.97	0.970	0.941	1.157	1.735	0.39	0.86
SIC 19	3.55	1.353	1.830	0.038	−0.692	0.40	0.86
SIC 20	3.19	1.402	1.967	0.335	−0.640	0.51	0.86

**Table 6:** Correlational analysis (Study 2).

	1	2	3	4	5	6
1. SIC	1					
2. SIC_Succession	0.85 **	1				
3. SIC_Consumption	0.79 **	0.45 **	1			
4. SIC_Identity	0.75 **	0.43 **	0.53 **	1		
5. FSA	0.60 **	0.38 **	0.61 **	0.51 **	1	
6. ASD	0.21 **	0.18 **	0.20 **	0.14 **	0.24 **	1

Note: ** *p* < 0.01.

**Table 7:** Gender multiple-group analysis.

	χ^2^	df	*p*	RMSEA	CFI	TLI	SRMR	AIC	ΔCFI	ΔRMSEA	ΔSRMR
Male	268.518	113	0.000	0.050 (0.04–0.06)	0.933	0.919	0.051	26,621.801			
Female	259.370	113	0.000	0.052 (0.04–0.06)	0.940	0.928	0.052	23,800.425			
Configural	528.160	226	0.000	0.051 (0.05–0.06)	0.937	0.924	0.051	50,422.226	-	-	-
Metric	551.353	240	0.000	0.051 (0.05–0.06)	0.935	0.926	0.056	50,419.459	0.002	0.000	0.005
Scalar	609.132	254	0.000	0.052 (0.05–0.06)	0.925	0.920	0.058	50,454.689	0.010	0.001	0.002

**Table 8:** Age multiple-group analysis.

	χ^2^	df	*p*	RMSEA	CFI	TLI	SRMR	AIC	ΔCFI	ΔRMSEA	ΔSRMR
Young	393.850	113	0.000	0.060 (0.05–0.07)	0.918	0.901	0.053	33,576.506			
Adult	179.034	113	0.000	0.043 (0.03–0.06)	0.945	0.934	094.	15,872.000			
Configural	564.269	226	0.0000	0.055 (0.05–0.06)	0.925	0.910	0.053	49,145.648	-	-	-
Metric	577.191	240	0.0000	0.053 (0.05–0.06)	0.925	0.915	0.056	49,131.415	0.000	0.002	0.002
Scalar	661.689	254	0.0000	0.057 (0.05–0.06)	0.910	0.903	0.060	49,199.183	0.015	0.004	0.004

## Data Availability

The data that support the findings of this study are available from the corresponding author upon reasonable request.
